# Image-Based Pain Intensity Estimation Using Parallel CNNs with Regional Attention

**DOI:** 10.3390/bioengineering9120804

**Published:** 2022-12-14

**Authors:** Xinting Ye, Xiaokun Liang, Jiani Hu, Yaoqin Xie

**Affiliations:** 1 Shenzhen Institute of Advanced Technology, Chinese Academy of Sciences, Shenzhen 518055, China; 2 University of Chinese Academy of Sciences, Beijing 100049, China; 3 Radiology Department, Wayne State University, Detroit, MI 48201, USA

**Keywords:** pain intensity estimation, parallel CNNs, regional attention, UNBC dataset

## Abstract

Automatic pain estimation plays an important role in the field of medicine and health. In the previous studies, most of the entire image frame was directly imported into the model. This operation can allow background differences to negatively affect the experimental results. To tackle this issue, we propose the parallel CNNs framework with regional attention for automatic pain intensity estimation at the frame level. This modified convolution neural network structure combines BlurPool methods to enhance translation invariance in network learning. The improved networks can focus on learning core regions while supplementing global information, thereby obtaining parallel feature information. The core regions are mainly based on the tradeoff between the weights of the channel attention modules and the spatial attention modules. Meanwhile, the background information of the non-core regions is shielded by the DropBlock algorithm. These steps enable the model to learn facial pain features adaptively, not limited to a single image pattern. The experimental result of our proposed model outperforms many state-of-the-art methods on the RMSE and PCC metrics when evaluated on the diverse pain levels of over 12,000 images provided by the publicly available UNBC dataset. The model accuracy rate has reached 95.11%. The experimental results show that the proposed method is highly efficient at extracting the facial features of pain and predicts pain levels with high accuracy.

## 1. Introduction

As one of the most common clinical symptoms, pain is a complex and subjective feeling. In 2020, the International Association for the Study of Pain (IASP) redefined pain as “An unpleasant sensory and emotional experience associated with or resembling that associated with, actual or potential tissue damage” [1]. Moreover, the former president of the American Pain Society, James Campbell, introduced the idea of “Pain as the 5th Vital Sign” (P5VS) in 1996. Specifically, pain is the fifth vital sign after the four vital signs of body temperature, pulse, respiration, and blood pressure. According to this concept, pain intensity should be regularly assessed, together with the classic four vital signs. Further, pain is not solely a sensory experience; it is more complex to assess, evaluate and manage than originally anticipated [2].

Accurate pain assessment is the first step in pain management and runs through the pain treatment. It can help aphasia patients to express the pain and discomfort caused by medical and surgical operations in real-time so that doctors can reasonably deal with the patient’s next medication control and treatment procedures. Therefore, quantifying the degree of pain plays an important role in medical and health care.

At present, in the medical field, scale evaluation and recording methods are mostly used to monitor the degree of pain. The most widely used evaluation tools include the visual analog scale (VAS), numeric rating scale (NRS), verbal rating scale (VRS), and faces pain scale-revised (FPS-R) [3]. In 2021, Daniel et al. [4] published a survey report on clinical pain monitoring, in which 109 hospitals participated. The most used scale for evaluating pain intensity in the hospitals was a numeric rating scale (81.6%), and most of the hospitals (73.5%) reported using combination scales, selecting the appropriate one according to the patient. The use of reliable and validated scales can improve routine pain management. However, these scales are more commonly used in measuring the degree of acute pain, not chronic pain [5] or severe patients [6]. Further, it was reported that nearly 15% were dissatisfied with their pain control [7]. Inaccurate pain assessment and the resulting inadequate treatment of pain in critically ill adults can have significant physiologic consequences [8]. There are difficulties in information communication in clinical pain management. For the effective expression of chronic pain patients, Dana et al. [5] developed a device and methodology for the noninvasive detection of nociception stimulation to improve the monitoring accuracy of chronic pain. For patients with pain relief through heavy anesthesia, anesthesiologists need intelligent computer systems to provide more physiological information to assist medication [9].

Therefore, it is known that in the case of unmanaged postoperative pain, the main causes are insufficient multidimensional pain assessment, delays between assessment and analgesic administration, inadequate use of analgesics (under or overmedication), and poor communication between clinicians and patients [2,10]. Meanwhile, unlike the classic four vital signs, the self-reporting of pain is a subjective measurement, and clinicians should accept patient reports and act upon them. In other words, these evaluation standards depend on an individual’s expression with large perceptual differences which leads to difficulties in accurate pain quantification.

In addition, the comprehensive quantification of the degree of pain based on physiological data is also often used in clinical supervision. This approach directly avoids the individual perceived differences caused by the abovementioned scale monitoring. However, the monitoring equipment is highly professional, requiring medical staff to operate the instrument, and the equipment is more expensive, which is not suitable for large-scale promotion. Meanwhile, the contact monitoring mode undoubtedly causes additional discomfort to the patient. For example, ECG signals are obtained by electrodes attached to the body, and the blood pressure also needs to be monitored by close-range sensors. Therefore, it is necessary to find a non-contact information acquisition method to alleviate the discomfort of the patient during their assessment.

Rich facial expressions can intuitively express the characteristics of personal physical and psychological emotional information. Compared with the language expression that also conveys information, it is more authentic. Therefore, facial expression has irreplaceable importance in pain monitoring. The research on the objective evaluation system of facial expressions started with the Facial Action Coding System (FACS) proposed by Ekman et al. [11] in 1978, which is based on the relationship between human expressions and facial muscle actions. In the branch of painful expression, Prkachin et al. [12] used FACS as a benchmark to create a PSPI (Prkachin and Solomon Pain Intensity) system to evaluate the pain level of facial expressions based on the corresponding state of the eyes, eyebrows, mouth, and other facial areas. Nevertheless, the PSPI evaluation system is based on action units (AUs), which require a lot of manpower to mark the video frames. It cannot realize actual clinical monitoring in real-time.

Early studies tended to design a dichotomous model that automatically distinguished pain from no pain. Ashraf et al. [13] used active appearance models (AAM) to decouple shape and appearance parameters from digitized painful-face images. Based on AAM, Lucey et al. [14] combined SVM to classify pain from no pain. According to the formulation of the PSPI [12] rule, people began to transform the task of pain detection from dichotomy to multilevels. Rizwan Ahmed Khan et al. [15] extracted shape information using a pyramid histogram of oriented gradients (PHOG) and appearance information using a pyramid local binary pattern (PLBP) to obtain a discriminative representation of the face. Based on the natural onset–apex–offset evolution pattern of facial expression, Zhao et al. [16] proposed an efficient optimization algorithm based on the alternating direction method of multipliers (ADMM) and applied it to the study of pain expression. Wang et al. [17] fine-tuned a state-of-the-art face verification network using a regularized regression loss and additional data with expression labels.

More recently, a few attempts have been made to estimate the facial pain level using deep neural networks. Zhou et al. [18] proposed an end-to-end real-time regression framework based on the recurrent convolutional neural network. Rodriguez et al. [19] used CNNs to learn facial features, linking to LSTM to exploit the temporal relationship between video frames. Tavakolian et al. [20] used several convolutional layers with diverse temporal depths to form a spatiotemporal convolutional neural network (SCN) for automatic pain analysis. Wang et al. [21] proposed a C3D network to extract spatiotemporal facial features and combined the HOG (Histogram of Oriented Gradient) feature histogram in 2D images as geometric information to distinguish the degree of pain in facial expressions. Mohammad et al. [22] proposed statistical spatiotemporal distillation (SSD) to encode the spatiotemporal variations underlying the facial video into a single RGB image and then used 2D models to process video data. Xin et al. [23] performed a 2D affine transformation (i.e., translation, cropping, rotation, scaling, and skewing) against background interference, spatial transformation, and attentional CNN to improve the estimation performance. Huang et al. [24] proposed a deep spatiotemporal attention model PAN (Pain-Attentive Network), which integrates the attention mechanism of both spatial and temporal dimensions, but this model consumed a lot of computing resources. Huang et al. [25] proposed an end-to-end hybrid regression network called HybNet, which integrates a 3D convolution network capturing spatiotemporal features, a 2D convolution network capturing spatial features, and a 1D convolution network capturing geometric features.

However, in previous studies, the pain dataset did not consider separating the foreground and the background in the painful expression image but directly imported it into the experimental model. Such an operation will lead to a difference in background scenes and can give wrong guidance in the training of the model, which affects the classification of the degree of pain. Moreover, in the practical clinical scene, the background is diversified by the presence of other objects which may have a negative impact on the evaluation of the degree of pain model. Some researchers [23] solved the above problems by clipping the image frame, but there is a time overhead and a lack of flexibility in the clipping operation which greatly depends on the pattern of the image sample. To put it simply, an algorithm can only solve one image pattern.

Therefore, in order to prevent the background information in the pain image data set from negatively affecting the model training, we introduced parallel CNNs, a parallel convolutional neural network with two branches. One branch focuses on the characteristics of the core regions, and the other supplements the missing information in feature transmission. The core regions are mainly based on the tradeoff between the weights of the channel attention modules and the spatial attention modules. Meanwhile, the background information in the non-core regions is shielded by the DropBlock algorithm [26]. These steps enable the model to learn facial pain features adaptively without being limited to a single image pattern.

## 2. Related Work

### 2.1. Convolutional Neural Network

In 2012, Alex Krizhevsky et al. achieved significant progress in ImageNet with AlexNet [27]. Then, the structure of the convolutional neural network began to diversify in terms of depth [28], width [29], and trick [30].

In 2014, Simonyan et al. proposed VGGNet [28] with a deeper network structure. It was shown that the superposition of the blocks with the same shape and smaller convolutional kernels performs well in feature extraction. Soon after, VGGNet was put into facial research. The VGGface working group [31] collected a large dataset from the Internet, trained the network structure, and then fine-tuned the network through a triplet loss function, eventually yielding an accuracy of 98.95%. This demonstrates the high applicability of VGG networks in facial structure recognition.

In 2016, Kaiming He proposed the residual structure applied to the ResNet [30] network which solved the model degradation and gradient disappearance. In 2017, SphereFace et al. [32] applied the ResNet to facial feature recognition and proposed a softmax (a-softmax) loss function to distinguish facial features with angular edges, increasing the performance on LFW to 99.42%.

### 2.2. Parallel CNNs

Compared with the single network structure, the parallel network structure can extract more abundant feature information to improve the generalization ability of the model. Therefore, it is widely used in the fields of object classification [33,34], object recognition [35,36], object detection [37], and so on.

Gao et al. [33] developed a two-column parallel network for RGB images and depth images to identify hand gestures. Wang et al. [34] applied Bilinear CNNs to the stain decomposition on histopathological images and obtained classification results superior to other single CNN structure models. Chowdhury et al. [35] applied Billinear CNNs to face recognition and showed substantial improvements over the standard CNN. Yang et al. [36] proposed the D-PCN framework consisting of two parallel CNNs, a discriminator, and an extra classifier which takes integrated features from parallel networks and gives a final prediction. Zhang et al. [37] organized deep and shallow CNNs in parallel structures, realizing the simultaneous detection of large and small objects.

### 2.3. Attention Mechanism

The attention mechanism is a signal-processing mechanism of the human visual perception system. Human vision can quickly scan the global image to get the target area that needs to pay more attention to get more details of the target and to suppress other useless information [38]. Then, a variety of special information extraction structures are drawn according to this working mechanism to automatically learn and calculate the contribution of input data to the output data. Attention mechanisms have proven to be useful in fields such as scene segmentation [39,40], image understanding [41,42], fine-grained visual classification [43,44], and image inpainting [45,46].

In the field of attention mechanism, the most representative spatial attention mechanism and channel attention mechanism [47] have been widely used in applied research in various fields. Chen et al. [48] proposed a deep object co-segmentation method based on channel and spatial attention, which combines the attention mechanism with a deep neural network to enhance the common semantic information of common objects. Liao et al. [49] proposed a channel–spatial–temporal attention-based network to refine and enrich the discriminative sample-specific features in three dimensions, namely, channel, space, and time simultaneously. Karthik et al. [50] proposed an ensemble of two CNN architectures integrated with channel and spatial attention to extract features in parallel and obtain performance improvements. Gohil et al. [51] proposed enhancements to the 3D U-Net model to incorporate spatial and channel attention in order to improve the identification and localization of segmentation structures by learning spatial context.

The face structure is universal so the external features and the texture details generated by the internal muscle movement are also extremely universal. Therefore, the attention mechanism has been widely used in isomorphic facial expression recognition. Chinnappa et al. [52] proposed focusing attention on the microexpression analysis, fusing the feature information of channel and space dimensions, to obtain the interspatial kindred matrix. Wang et al. [53] proposed a new anchor-level attention, highlighting the features from the face region.

To sum up, the research applied in different fields also supports the strong robustness, generalization, and universality of spatial attention mechanisms and channel attention mechanisms from different perspectives.

In previous pain monitoring work with attention mechanisms, feature extraction typically highlights pain features while ignoring the rest [24,25] or extracts the facial area through traditional image editing methods [23]. In our work, the non-core regions are determined according to the attention weights. Afterward, DropBlock operations are performed for the non-core area.

## 3. Methods

The dataset used in this experiment belongs to the open-source UNBC-McMaster Shoulder Pain Expression Archive Database [54], which was jointly collected by McMaster University and the University of Northern British Columbia. Written informed consent was obtained from all participants before the study. As a third-party researcher, we have signed a “PAIN DATABASE USER AGREEMENT” with the dataset publisher. In this experiment, all the pictures used met the ethics standards.

### 3.1. Parallel CNNs

The parallel CNNs model uses two parallel CNN models, ResNet and VGGNet, but without the last fully connected softmax layer. We then combine two one-dimensional feature vectors into a one-dimensional feature vector by bilinear operation [55]. Following that, the softmax layer is used as the final linear classifier.

Parallel CNNs for facial expression classification consist of a quadruple $Q=\left(f_{A},f_{B},P,C\right)$. Here, $f_{A}$ and $f_{B}$ are feature extraction functions based on CNN, P is a fusion function, and C is a classification function. The feature function is a mapping, $f:\;L\times I\to R^{K\times D}$, that takes an image I∈I and a label l∈L and outputs a feature map of size K×D. Then, the two feature maps obtained by parallel CNNs are fused into one feature map through the operation of P. P is the bilinear operation [55]:

(1) $$\mathrm{Parallel}\left(l,I,f_{A},f_{B}\right)=f_{A}{\left(l,I\right)}^{T}f_{B}(l,I)$$

Through the specific models used in our experiments, we aimed to extract different features from the pain image data through CNNA and CNNB. We set VGG16 as the backbone network for CNNA and used it to extract the feature information in the core region emphatically. Commonly, it is difficult to accurately obtain the complete features due to the disadvantage of information loss in the feature transmission of deep networks. Moreover, the loss of feature information leads to a gradient disappearance or gradient explosion at a later stage. To avoid this, we take a ResNet network containing a shortcut connection as CNNB to compensate for the loss of feature information, which acts as a feature supplement.

The emphasis of the experiment was to extract feature information of the core area in branch A, which depends on the translation invariance of the CNN. It is, therefore, necessary to consider the performance of each network in translation invariance.

According to previous studies [56,57], max pooling has significant performance, although it is a small change. To overcome the sensitivity of max pooling, Zhang et al. [58] proposed the BlurPool method to improve the translation invariance of the network in 2019. In most cases, downsampling methods such as max pooling, strided convolution, and average pooling ignore the sampling theorem, which leads to the network losing the translation invariance, while the BlurPool method can reduce the aliasing effect without removing the maximum pool and make the network invariant. The BlurPool method specifically decomposes pooling sampling into two steps: first, taking the maximum value, and then downsampling; second, using the convolution low filtering operation to slow down the acquisition of the maximum value.

**Figure 1:**
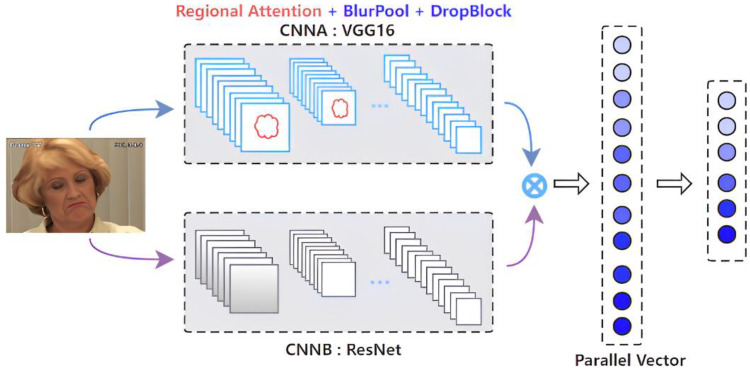
Block diagram of our proposed methodology.

The network structure of CNNA requires a higher translation invariance when extracting information from the network, so we used BlurPool for feature extraction. The specific network structure is shown in Figure 1.

### 3.2. Insertion of the Attention Mechanism

The attention mechanism is essentially similar to the human observation behavior of external things. Generally speaking, when people observe external things, they first pay more attention to meaningful local information and then combine the information from different regions to form an overall impression. The attention mechanism can help the model assign different weights to each part of the input X, extract more critical information, and make a more accurate judgment without bringing more overhead to the calculation and storage of the model. Therefore, we applied the attention mechanism to the deep learning network so that our network model could automatically learn and obtain the facial expression data from diverse input data.

Considering that CNNA networks need to have efficient feature extraction capabilities, we can apply the channel and spatial attention mechanism to CNNA to extract facial expression feature information more accurately.

#### 3.2.1. Spatial Attention

Our facial pain expression data set has a robust programming structure. In order to better obtain facial pain expression features and make the model more robust, we applied the attention mechanism guided by feature extraction from the spatial domain.

**Figure 2:**
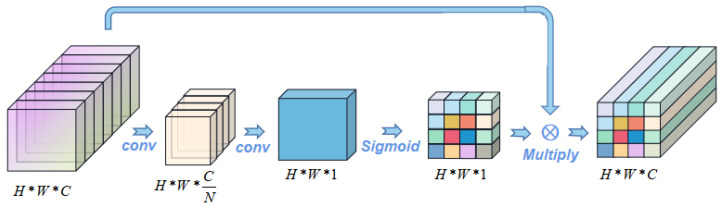
Spatial attention mechanism.

The spatial attention mechanism [47] carries out the corresponding spatial transformation of spatial domain information in the image, generates the mask and scores the space, and then extracts the key information. It is similar to adding weight to the signal on each pixel to represent the correlation between the pixel value and key information. The larger the weight is, the higher the correlation is. In other words, according to the cross-section of the H*W*C feature map, data compression is performed pixel by pixel. The compression of 1*1*C data is transformed into 1*1*1 compression features and the feature information of H*W is synthesized as the mask, and then scaled back to the original H*W*C feature map, as shown in Figure 2.

#### 3.2.2. Channel Attention

Through the spatial attention mechanism in the previous step, we can obtain facial pain expression features more efficiently. However, the information in the channel domain is ignored. The image features in each channel are treated equally, limiting the spatial domain transformation method to the original image feature extraction stage, without strong interpretability of the application in other layers of the neural network layer. In order to make the model more efficient and flexible in feature extraction, we apply the channel attention mechanism guided by feature extraction in the channel domain.

**Figure 3:**
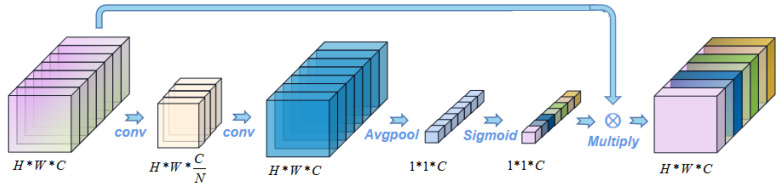
Channel attention mechanism.

The channel attention mechanism [47] was used to extract the channel domain information in the image. Channel weights were generated and scored. It is similar to adding weight to the signal on each channel to represent the correlation between the channel and key information. The larger the weight is, the higher the correlation. In other words, for the channels of the H*W*C feature map, data compression is carried out one by one on cross-sections, and the feature data of H*W*1 is compressed and transformed into 1*1*1 compression features. The feature information of 1*1*C is then synthesized as the channel weight, and then acted back to the original H*W*C feature map, as shown in Figure 3.

### 3.3. Enhancement of Features

To enable a better extraction of facial pain features in later experiments, it is necessary to divide the experimental video frame into a foreground and a background area. That is, an image mask is generated to divide the foreground and background areas, to facilitate the subsequent filtering of features in background areas to enhance the relevant features.

The reinforcement of correlated features is equivalent to the weakening of unrelated features. The critical processing processes can be reduced to:

1. The selection of low-correlation channels;
2. The segmentation of regions with low correlation;
3. The enhancement of the high-correlation features.

#### 3.3.1. Selection of Low-Correlation Channels

Based on the working principle of the convolution layer of CNN, the information extracted from the image still has spatial correlation, and there are channels in the network that have a greater degree of correlation with the foreground. Obviously, we can improve the high-correlation feature extraction rate by reducing the features of low-correlation channels.

To the best of our knowledge, gradient-weighted class activation mapping (Grad-CAM) [59], is an example-based explanation method that provides a gradient activation heat map as an explanation for convolution neural network (CNN) models. That is, to color the 2D feature map with different color blocks. The weight of a larger gradient is represented by a warmer color (e.g., Red), and the weight of a smaller gradient is represented by a cooler color (e.g., Blue). In this way, warm-colored areas can be used to represent the corresponding feature map that receives more attention during training.

**Figure 4:**
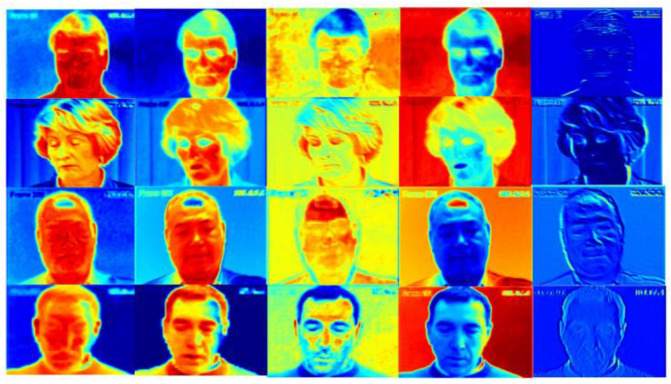
Heatmap for the channels with decreasing weights in the CA module. The change in color from warm to cold (e.g., red to blue) represents the reduction in attention received during training.

Therefore, we compared the C*1*1 channel weight information, the output of the CA (Channel Attention) module, with the heatmap under the corresponding channel, and noticed that the channels with the higher weights applied most of the computational resources to the focused facial expression regions, as shown in Figure 4. Therefore, the channel with the lower weight of the CA module output was selected as the filter channel, where fewer facial pain expression features exist.

We determined the selection of filtering channels based on the quartile rule. The mathematical language represents the output of the weight by the CA module regarding the channel-by-channel weights, where $c_{i}$ represents the weight of the i-th channel. Then, according to the weight size of the ascending sort, we get $C^{\prime }=\left[c_{1}^{\prime },c_{2}^{\prime },\ldots c_{n}^{\prime }\right]$. According to the quartile rule, the position of the first quartile Q1 was calculated in Equation (2), where n represents the number of weights or the number of channels. Then, we can determine the weights of all values from zero to the top 25%. The channel corresponding to the first 25% weight is masked, which is the filter channel we selected (Equation (3)).

(2) $$Q_{1}=\left(n+1\right)\times 0.25$$

(3) $$C_{\mathrm{filter}}=\left[c_{1}^{\prime },c_{2}^{\prime },\ldots c_{Q_{1}}^{\prime }\right]$$

#### 3.3.2. Segmentation of Regions with Low Correlation

**Figure 5:**
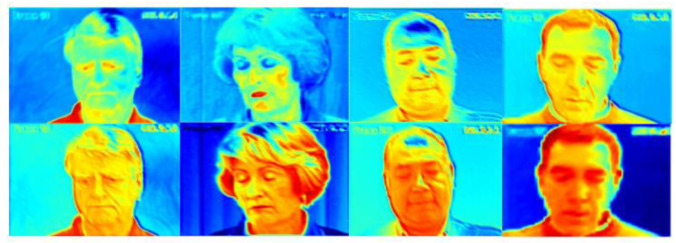
Comparison of the heat map before and after the SA module. The change in color from warm to cold (e.g., red to blue) represents the reduction in attention received during training.

After determining the filter channels with a low relationship with the foreground target, it was necessary to find the location of the features associated with the foreground target from the filter channels to divide the core region and the non-core region. After the SA (Spatial Attention) module, the computational resources will focus on the foreground information. The specific heat map generated by the Grad-CAM algorithm [59] is shown in Figure 5. The neurons with high weight have more of an influence on the target task. We set the weight located at the first quartile Q1 of the output in the SA module as the threshold.

The output of the SA module is the 2D weight information of H*W. To rank its weight information, H*W is converted into a 1D vector of 1*(H*W). According to the quartile rule, the position of the first quartile Q1 was calculated in Equation (2), where n is the value H*W, which is the number of pixels in any channel layer. Therefore, the threshold is the weight at the Q1 location in the sorted 1D vector.

Generally speaking, the unit which is larger than the threshold is regarded as the neuron associated with the task target. In contrast, the correlation degree is low. A set of neurons whose weight is higher than the threshold is identified as the core area in the foreground target. s(x,y) represents the weight of position (x,y) of the output in the SA module.

**Figure 6:**
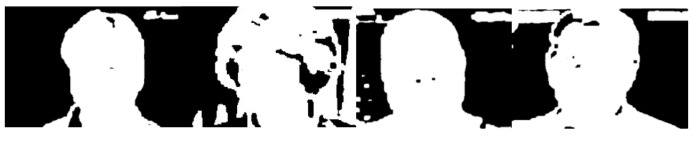
The binary video frame mask.

Meanwhile, based on the spatial invariance of CNN, neurons in other channels in the core area are still related to the foreground target. In other words, although different channels will extract the feature from different focus points, the information retained by neurons in the same position on different channels still comes from the same area on the image. Therefore, we should make a video frame mask to obtain the features of the core area, as shown in Figure 6. The binary video frame mask is generated as follows:

(4) $$\mathrm{mask}\left(i,j\right)=\{\begin{array}{ccc} 0 & s\left(x,y\right)\geq w_{k} & \left(x,y\right)\in U_{\mathrm{focus}}\\ 1 & s\left(x,y\right)<w_{k} & \left(x,y\right)\notin U_{\mathrm{focus}} \end{array}$$

In the filter channel obtained from the previous step, by multiplying the binary mask with the corresponding feature map, the neurons in the core region will be retained, but the neurons in the non-core region will be shielded. To get the opposite result, just invert the binary mask and repeat the above operation.

#### 3.3.3. Enhancement of the High-Correlation Features

The model should focus on the pain expression in the foreground when extracting the feature information, ignore the irrelevant information such as the background, and improve the efficiency of the information. For this reason, we introduced the DropBlock algorithm into the early stage of feature extraction, i.e., the regional regularization of shallow data.

Dropout [60] is a practical technique to mitigate overfitting in the work of fully connected neural networks, which reduces the activation of neurons at a fixed rate during training. In the previous experiments on facial expression, dropout is often used to regularize the extracted feature, but this operation has little effect on the convolution layer with spatial information. In 2018, Google Brain researchers [26] proposed a DropBlock algorithm targeting convolutional layer regularization. This method can effectively reduce local semantic information in the convolutional layer, stimulate the network to learn more robust and effective features, and avoid the adverse effects of random feature loss.

The pseudo-code of the DropBlock [26] algorithm combined with area division is as follows:
**Algorithm 1:***Mask Generation Based on DropBlock***Input:**$C=\left[c_{1},c_{2},\ldots ,c_{n}\right],mode;$    1:   if mode==Interface then    2:   $return\;C^{*}=C$    3:   end if    4:   $The\;average\;activation\;value\;of\;each\;c_{i}\;was\;calculated\;as\;w_{i},\;and\;the\;c_{k}\;with\;the\;largest\;w\;was\;obtained;$    5:   $Get\;c_{k}\left(\bar{x},\;\bar{y}\right)\;as\;the\;unit\;with\;the\;highest\;activation\;value;$    6:   $Make\;all\;zero\;mask\;matrix\;M\;the\;same\;size\;as\;c_{i};$    7:   for each m(i, j) in m do    8:    $if\;c_{k}\left(i,j\right)>random\left(0.6,\;0.9\right)\cdot c_{k}\left(\bar{x},\;\bar{y}\right)\;then$    9:       m(i,j)=1;     10:      end if    11:  end for    12: $\;return\;C^{*}=C\;⊙M\;$

The core parameters in the DropBlock algorithm are block_size and γ. The block_size represents the size of the shielded area. When block_size = 1, the DropBlock algorithm is equivalent to the ordinary dropout algorithm. The parameter γ represents the probability that the activated unit will be blocked. The specific calculation formula is as follows:

(5) $$\gamma =\frac{1-\mathrm{keep\_prob}}{\mathrm{block\_size}}\frac{{\mathrm{feat\_size}}^{2}}{{\left(\mathrm{feat\_size}-\mathrm{block\_size}+1\right)}^{2}}$$

## 4. Experiments

### 4.1. Preparation of UNBC

The dataset used in this experiment is the open-source UNBC-McMaster Shoulder Pain Expression Archive Database [54], and we take the PSPI scoring system [12] to objectively score the experimental image data as ground truth. Two hundred sequences of 25 subjects were recorded (48,398 frames in total). For each frame, discrete pain intensities (0–15) according to Prkachin and Solomon are provided by the database creators. The Prkachin and Solomon Pain Intensity (PSPI) metric, which measures pain as a linear combination of facial action units, is shown in Equation (6). This scoring system subdivides pain expressions into 16 scales.

(6) PSPI=AU4+max(AU6;AU7)+max(AU9;AU10)+AU43

According to the Prkachi–Solomon Pain Intensity (PSPI) pain metric, the researcher encoded pain levels for each frame of the entire UNBC dataset [54]. Level 0 is the natural expression in the pain-free stimulation state, and correspondingly, the highest grade of 15 is the highest pain level, with severe deformation of the facial unit. The frequency of each pain intensity is summarized in Table 1.

To solve the problem of data dispersion shown in Table 1, most of the previous studies adopted the clustering method, merging the classification from 16 scales to 6 scales. The original 16 pain levels are discretized in a data-balancing manner. Specifically, levels 4 and 5 are merged and pain levels 6 and above are categorized as one class. Then we get a more significant data set, which also has a positive effect on the training of the model. The cluster-adjusted frequencies are also shown in the last column of Table 1.

**Figure 7:**
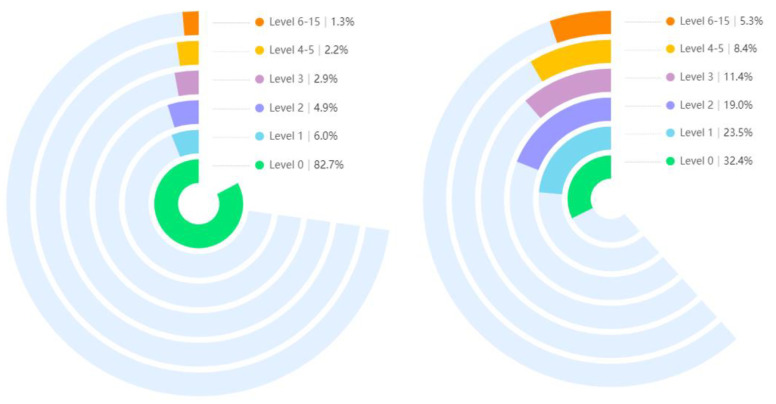
Comparison of data volumes for the six pain levels after clustering.

To solve the larger level of level 0 after clustering, we take one of every ten of the image frames in level 0, just selecting 1/10 of the data to balance the data distribution. The proposed integration operation is shown in Figure 7. By randomly selecting, the proportion of natural expression frames was reduced from 82.7% to 32.4%, making the distribution of the whole dataset more balanced.

### 4.2. Experiment on UNBC

To support the comparison with previous work, we used the root mean square error (RMSE) and Pearson correlation coefficient (PCC) for performance evaluation. RMSE is computed as the difference between the predictions and the ground truth which are the PSPI annotations for each frame of pain expression. Similarly, the PCC measure evaluates the sequential prediction results of the model, equivalent to the time progression of pain expression.

(7) $$\mathrm{RMSE}=\sqrt{\frac{1}{N}\overset{N}{\underset{i=1}{\sum }}{\left({\hat{y}}_{i}-y_{i}\right)}^{2}}$$

(8) $$\mathrm{PCC}=\frac{\sum_{i=1}^{N}\left({\hat{y}}_{i}-\overset{-}{{\hat{y}}_{i}}\right)\left(y_{i}-\overset{-}{y}\right)}{\sqrt{\sum_{i=1}^{N}{\left({\hat{y}}_{i}-\overset{-}{{\hat{y}}_{i}}\right)}^{2}}\sqrt{\sum_{i=1}^{N}{\left(y_{i}-\overset{-}{y}\right)}^{2}}}$$

Here, N is the total number of frames of videos, $y_{i}$ and ${\hat{y}}_{i}$ are the ground truth and the predicted pain results of the i-th frame, respectively. Similarly, $\overset{-}{{\hat{y}}_{i}}$ and $\overset{-}{y}$ are the mean of the corresponding $\left\{{\hat{y}}_{1},{\hat{y}}_{2},\ldots ,{\hat{y}}_{N}\right\}$ and $\left\{y_{1},y_{2},\ldots ,y_{N}\right\}$.

#### 4.2.1. Ablation of the Attention Modules in the CNNA

In the CNNA structure of parallel CNNs, we integrate the channel attention module and spatial attention module. Then, we can make the foreground feature extraction model more efficient to extract the facial expression feature information more accurately.

For the effectiveness and location arrangement of the two attention modules, we performed the following experiments to specifically assess the appropriate manner. First, we separately validated the positive performance improvement of the two attention modules, and later, compared the specific arrangement of the two modules, including sequential CA–SA and sequential SA–CA.

The experimental results of different CNN modules are summarized in Table 2, which are obtained after adding different attention modules to branch A of the parallel CNNs. ”CA” indicates that the channel attention is placed alone on branch A in parallel CNNs. “SA” indicates that the spatial attention is placed in the same position as “CA”. “CA–SA” indicates that the channel attention submodule and spatial attention submodule are placed into branch A in serial order. “SA–CA” indicates that it is placed in the same position in the reverse order of “CA–SA”.

Table 2 shows that both modules have made a positive performance improvement in the model. The combination of CA–SA achieves the best performance improvement, which is consistent with the findings of the CBAM block [47].

#### 4.2.2. Ablation of the Model Structure

For our final parallel CNNs model, we performed two sets of experiments to evaluate our proposed framework. First, we compared three different network structures: single modified CNNA, single CNNB, and parallel CNNs with both modified CNNA and CNNB. As each CNN module has a different structure, and the structure determines what features are extracted. In this case, both CNN modules can be applied in parallel, then the outputs of the two CNN modules are added by bilinear operation [55] and normalized with the softmax function.

**Figure 8:**
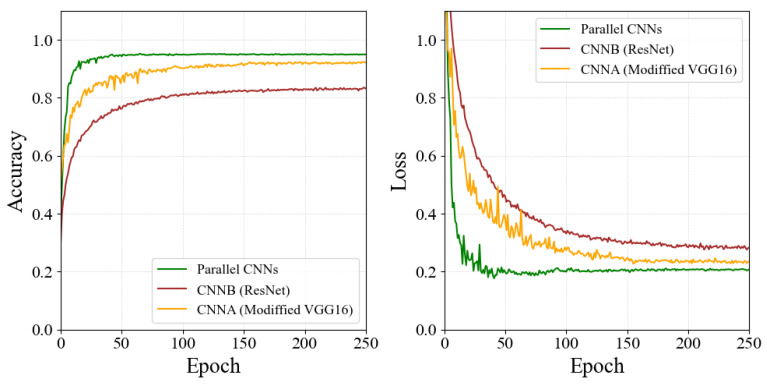
Plots for the convergence in the accuracy and loss of different CNN modules with their composite structures.

To demonstrate the effectiveness of the model, we did the same experiment on the framework of parallel CNNs and their two separate branch networks. The accuracy and loss convergence plots for the model training are shown in Figure 8, and the quantification results of the RMSE and PCC indicators performed on the models are also shown in Table 3.

Based on the analysis of the experimental results, we can see that the extracted facial pain features based on the CNNA branch contribute more to the classification accuracy of the whole network. Note that the modified CNNA module performs much better than the CNNB module. Surprisingly, the fusion of the feature vectors extracted from the CNNA and CNNB branches resulted in higher accuracy, a higher PCC, and a lower RMSE in the pain intensity estimation. In other words, the parallel method outperforms any single CNN module, showing that it is crucial to utilize the feature map extracted by different CNN modules at the same time while the best-arranged strategy further improves the performance. This result also supports the robustness and effectiveness of the model.

#### 4.2.3. Comparison with Other State-of-the-Art Methods

It should be noted that to improve the quality of the data in this experiment, the category of pain expression was reduced from the original PSPI 16-point problem to a 6-point scale. As a result, we selected state-of-the-art methods that also used the same data clustering processing rule for comparison. As shown in Table 4, our model performs well with regard to RMSE and PCC.

**Figure 9:**
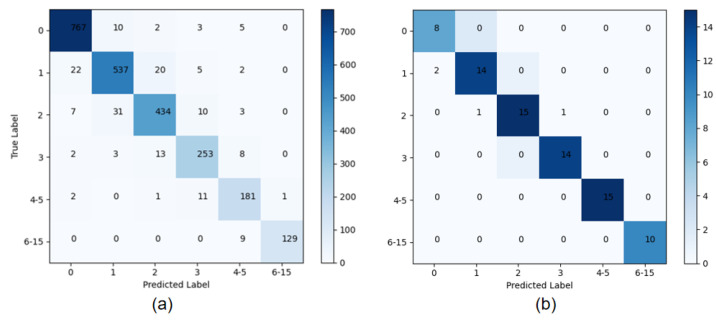
Confusion matrix for the prediction results of pain frames based on Parallel CNNs. (**a**) shows the confusion matrix for the predicted results of the whole test dataset, and (**b**) shows the confusion matrix for the predicted results of an 80-frame video.

We can obtain a more balanced dataset based on the original dataset. The dataset was then divided into training and test sets by an 8:2 ratio. After the model training, the model was performed on the test dataset to test the effectiveness of the model. The confusion matrix of the result is shown in Figure 9a.

The confusion matrix shows that the overall accuracy is 95.11%. Among them, the poor performance is mainly the detection among level 1 and level 2, and the better performance is level 0 and levels 6–15. It can also be seen that despite the data imbalance, the accuracy of levels 6–15, with the smallest amount of data, is not below average. This clearly shows that our model has higher pain recognition accuracy for high levels and satisfies practical applications.

**Figure 10:**
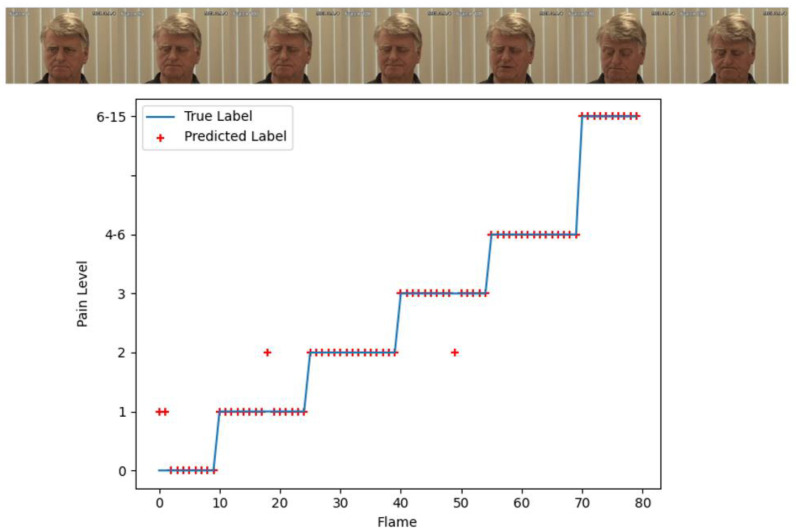
The comparison of the prediction and ground truth for an 80-frame video with pain expression.

To show the experimental results more clearly, we selected one pain video for a visual visualization of the experimental results. The confusion matrix and the comparison of the prediction and ground truth are shown in Figure 9b and Figure 10. The video has 80 frames. There are only four frames of prediction errors and they mainly focus on the early start stage of the pain. The figures clearly show that our model has higher pain recognition accuracy for high pain levels and satisfies practical applications.

## 5. Conclusions

Accurate pain assessment is the first step in pain management and runs through the pain treatment. We proposed parallel CNNs to focus on learning core regions while supplementing global information, which enables the adaptive extraction of pain features in the face area to not be limited to a single image pattern. In particular, feature learning in the core regions is mainly based on the relationship of the output of the weight by the channel and spatial attention modules. At the same time, the background information of the non-core region is shielded by the DropBlock algorithm.

We applied this improvement to the UNBC datasets to test the performance of the model and it outperformed most state-of-the-art methods based on the RMSE and PCC metrics. The proposed method improves the efficiency of facial expression pain feature extraction and makes the prediction of pain level more accurate.

However, our proposed method is image-based and the utilization of the dataset is limited by unbalanced label distribution. Therefore, in further future work, we intend to combine the time-series feature information with a more fine-grained pain intensity estimation, and an appropriate method to improve the utilization of the dataset will be sought.

## Figures and Tables

**Table 1:** The frequency of each pain intensity in the UNBC dataset according to the Prkachin–Solomon Pain Intensity (PSPI) pain metric.

PSPI Score	Total Frames	Frequency	Frequency After Clustering
0	40,029	82.7439%	82.7439%
1	2909	6.0132%	6.0132%
2	2351	4.8597%	4.8597%
3	1412	2.9187%	2.9187%
4	802	1.6578%	2.1581%
5	242	0.5002%	
6	270	0.5581%	1.3498%
7	53	0.1096%	
8	79	0.1633%	
9	32	0.0661%	
10	67	0.1385%	
11	76	0.1571%	
12	48	0.0992%	
13	22	0.0455%	
14	1	0.0021%	
15	5	0.0103%	

**Table 2:** Comparison of different attention modules with their composite structures.

Attention Combination	RMSE	PCC
CA	1.02	0.74
SA	1.13	0.68
**CA–SA**	**0.73**	**0.85**
SA–CA	0.91	0.75

**Table 3:** Comparison of different CNN Modules with their composite structures.

Model Combination	RMSE	PCC
CNNA: Modified VGG16	0.73	0.85
CNNB: ResNet	1.15	0.67
**Parallel structure (Ours)**	**0.45**	**0.96**

**Table 4:** Comparison of the proposed approach with other state-of-the-art methods.

Description	RMSE	PCC
CRVR + (LBP and DCT) [61]	1.18	0.59
HoT [62]	1.10	0.53
RCNN [19]	1.24	0.64
LSTM [20]	0.86	0.78
BORMIR [63]	1.17	0.61
SCN [21]	0.57	0.92
PAN [25]	0.46	0.89
HybNet [26]	0.87	0.82
Parallel structure (Ours)	0.45	0.96

## Data Availability

The dataset used in this paper is the UNBC—McMaster Shoulder Pain Expression Archive Database, which is publicly available. We have signed the ”PAIN DATABASE USER AGREEMENT” with the relevant issuer.
